# Subtilases: a major prospect to the genome editing in horticultural crops

**DOI:** 10.3389/fpls.2024.1532074

**Published:** 2025-01-07

**Authors:** Umashankar Chandrasekaran, Woo Jong Hong, Hyeran Kim

**Affiliations:** ^1^ Institute of Life Sciences, Kangwon National University, Chuncheon, Republic of Korea; ^2^ Department of Biological Sciences, Kangwon National University, Chuncheon, Republic of Korea; ^3^ Department of Smart Farm Science, Kyung Hee University, Yongin, Republic of Korea

**Keywords:** protease, tomato, pepper, solanaceae, immunity, fruit ripening, abscission

## Abstract

Plant peptides, synthesized from larger precursor proteins, often undergo proteolytic cleavage and post-translational modifications to form active peptide hormones. This process involves several proteolytic enzymes (proteases). Among these, SBTs (serine proteases) are a major class of proteolytic enzymes in plants and play key roles in various regulatory mechanisms, including plant immune response, fruit development and ripening, modulating root growth, seed development and germination, and organ abscission. However, current knowledge about SBTs is largely limited to *‘in vitro cleavage assays*,’ with few studies exploring loss of function analyses for more in depth characterization. Research focused on economically significant horticultural crops, like tomato and pepper, remains scarce. Given this, leveraging SBTs for horticultural crop improvement through advanced gene-editing tools is critical for enhancing crop resilience to stress and pathogens. Over the past five years, research on proteolytic enzymes, especially SBTs, has increased markedly, yet reports involving loss- or gain-of function analyses aimed at improving crop yield and quality are still limited. This review summarizes recent findings on SBT enzymes, which act as ‘protein scissors’ in activating peptide hormones, and discusses the potential for using selected SBTs in CRISPR-Cas9 gene editing to enhance the growth and resilience of economically important Solanaceae crops, with a focus on pepper.

## Introduction

Gene expression and transcription are fundamental processes that ensure the effective functioning of multiple cellular systems throughout a plant’s life. Similarly, *de novo* protein synthesis (translation of a gene) and subsequent proteolytic degradation of protein regions, which are essential for its activity, are critical for plant growth and its response to environmental stimuli (Zhang et al., 2023; Son and Park, 2023). Several truncated protein peptides undergo post-translational modifications that regulate cell-to-cell communication, functioning as peptide hormones (Stührwohldt and Schaller, 2019). Over the past five years, there has been substantial progress in identifying novel signaling peptides and elucidating the mechanisms of peptide perception and signal transduction pathways (Hussain et al., 2021; Ghorbani et al., 2015; Kim et al., 2021; Pandita et al., 2023). The functional mechanisms for most of the identified plant peptide hormones are now well established (Royek et al., 2022). However, the biogenesis of these signaling peptides remains poorly understood, particularly in terms of identifying proteolytic cleavage sites and the utilization of proteolytic enzymes (proteases) in crops.

Some proteases are highly specific in their target site recognition, while others are more non-specific, hydrolyzing protein substrates into shorter peptides when conditions permit (Luciński and Adamiec, 2023). Specifically, SBTs (Pfam00082), which are serine proteases belonging to the S8 family (MEROPS database; https://www.ebi.ac.uk/merops/cgi-bin/famsum?family=S8), exhibit specificity in their proteolytic activity and play critical roles in processes such as plant immune response, fruit development, regulation of floral timing, root growth, adaptation to environmental changes and organ abscission (Falkenberg et al., 2022). However, much remains unknown about their divergence and evolutionary patterns across plant systems, with available information primarily limited to model plants like Arabidopsis and tomato (Reichardt et al., 2018; Matsui et al., 2024). With the recent discovery of a subtilase (SlPhy2) linked to drought-induced flower abscission in tomato, the need to identify SBTs in other Solanaceae crops has become increasingly important. However, knowledge regarding the identification and functional characterization of these proteases in other plants, especially economically important crops, remains limited (Reichardt et al., 2018; Figueiredo et al., 2016; Jin et al., 2021; Norero et al., 2016; Reichardt et al., 2020). Furthermore, most of these studies have focused solely on assessing SBTs as peptide cleaving enzymes through ‘*in vitro cleavage assays*’ (Xu et al., 2013; Royek et al., 2022). Consequently, studies addressing the functional characterization of these proteases through loss- or gain-of function approaches remain scarce.

Although the available information is limited, it underscores the importance of SBTs in plant growth and development. The AtSBT3.8-phytosulfokinane (PSK) and SlPhy2-PSK substrate activity are valid examples (Stührwohldt et al., 2021; Reichardt et al., 2020). Briefly, proteolytic processing of proPSKs into peptide hormone PSK by SBTs enhances root growth under abiotic stress, such as drought, and regulates organ abscission. This process primarily involves modulating cell expansion *via* a plasma membrane-localized module containing leucine-rich repeat receptor kinases (Li et al., 2024). Relatively, over-expression of a SBT gene AcoSBT1.12 has caused a delayed flowering time in pineapple (Jin et al., 2021). Moreover, an increasing application of SBTs (in the form of subtilisins) for the agricultural applications have also gained interest recently (Falkenberg et al., 2022). Given their involvement in plant immunity, stress regulation, agricultural applications, and abscission, the advancement of genetic tools like CRISPR-based editing techniques and whole genome analyses offers promising opportunities for further research and application. CRISPR genome editing technology has become a pivotal tool for plant breeders, enabling the development of improved cultivars with desired traits. It is widely applied across various aspects of plant research to produce precisely improved crops (Kafle, 2023; Yang et al., 2024).

In summary, this review highlights recent progress in the identification of plant SBTs and emphasizes potential SBT candidates as CRISPR targets to improve yield and quality in horticultural crops. While SBT members have been characterized in tomato, potato, and tobacco (Reichardt et al., 2018; Norero et al., 2016; Navarre et al., 2012), a complete catalog for pepper is lacking. Here, we present a comprehensive list of 91 SBTs identified in pepper through homology-based searches using closely related Solanaceae species, notably tomato along with their phylogenetic comparisons (**Supplementary Figure S1**, **Supplementary Table S1**).

## Active roles of SBTs in plant immune response

**Figure 1:**
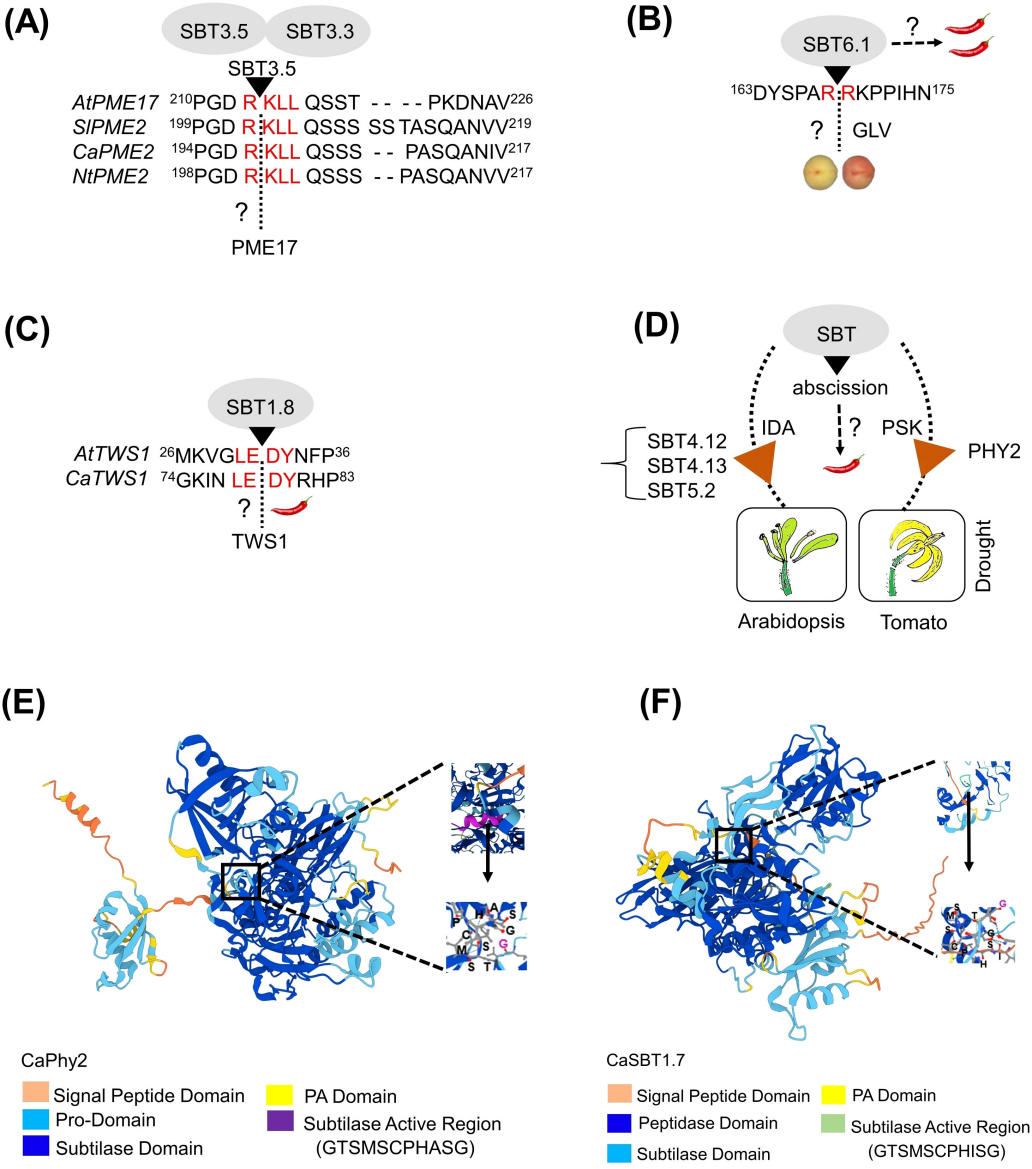
Illustrations of SBTs discussed in this review. **(A)** Cleavage of Arabidopsis pectin methylesterases (AtPME17) is shown. Multiple sequence alignment showed the conserved ‘R(R/K)LL’ motif among PME genes of Solanaceae family (tomato, pepper and tobacco). The possible role for SBT3.5 in cleaving the PME protein at the ‘R(R/K)LL’ motif among Solanaceae family genes is highlighted. **(B)** Proteolytic cleavage of proGLV at the ‘RRKP’ motif by SBT6.1 involving the peptide signaling towards root cell elongation. A possible role for SBT6.1 in cleaving proGLV during peach ripening is highlighted. **(C)** Cleavage of Arabidopsis Twisted Seed1 (TWS1) is shown. Sequence alignment showed the conserved ‘LEDY’ motif among TWS1 in both Arabidopsis and pepper. **(D)** Involvement of SBTs in floral organ abscission and flower drop in tomato is shown. Multiple SBTs like SBT4.12, SBT4.13 and SBT5.2 are involved in Arabidopsis floral organ drop, whereas only SlPhy2 subtilase is involved in flower drop during tomato drought stress. SBTs involved in organ abscission in pepper is highlighted. **(E, F)** Tertiary structure of CaPhy2 and CaSBT1.7 showed the ligand binding peptide residues in the active site like GTSMSCPHASG for CaPhy2 and GTSMSCPHISG for CaSBT1.7. The structures were created using alpha fold 3-web tool, https://deepmind.google/technologies/alphafold/.

Evolutionary changes and functional diversification have led to the acquisition of novel, plant-specific functions within the SBT family, contributing to its present-day complexity (Schaller et al., 2018). One example is the SERINE RICH ENDOGENOUS PEPTIDES (SCOOPs), a family of phytocytokines that are transcriptionally induced during immune responses in plants (Yang et al., 2023). In Arabidopsis, the pro-peptides (PROSCOOP) of SCOOP proteins are cleaved by multiple subtilases, including AtSBT3.3, AtSBT3.4, AtSBT3.5, AtSBT4.12, AtSBT4.13, AtSBT5.2, and AtSBT6.1, all identified through cleavage assays. However, functional studies to elucidate the roles of these SBTs in immune response remain scarce, with the exception of AtSBT3.5 (AT1G32940), which demonstrated a strong affinity for cleaving PROSCOOP peptides in a transient expression assay in Nicotiana benthamnia (Yang et al., 2023). Interestingly, in the same study, loss-of-function analysis using the sbt3^octopule^ mutant revealed a phenotype similar to that of the *mik2* mutant. MIK2 serves as the membrane-bound receptor for SCOOP peptides and plays a key role in triggering immune and stress responses, including resistance to herbivores, in *Arabidopsis* (Hou et al., 2021). Given the role of MIK2 gene in immune signaling, it would be reasonable to further analyze AtSBTs using advanced CRISPR-Cas9 tools. This approach enables the development of disease-resistant traits through targeted genetic modifications (**Figure 1A**).

The role of SBTs in immune responses, particularly in pathogen resistance, has been explored narrowly but in various plant species. *Arabidopsis* has focused primarily on AtSBT3.3 and AtSBT5.2 so far, while tomato have been investigated two SBT genes, SlP69B and SlP69C (Coculo et al., 2023; Ramírez et al., 2013; Zhang et al., 2024). AtSBT3.3 knockout and over-expression alters the salicylic acid (SA) mediated defense genes, thereby showing the sensitivity and resistance to bacterial pathogen *Pseudomonas syringae* DC3000 (Ramírez et al., 2013). Notably, AtSBT3.3 is the only *Arabidopsis* SBT gene associated with plant-bacterial pathogen defense mechanisms and is part of a genomic cluster containing three other SBTs: AtSBT3.2, AtSBT3.4, and AtSBT3.5. On the other hand, AtSBT5.2 interacts with transcription factor MYB30 to induce defense response against the bacterial pathogen *Pseudomonas syringae*. Arabidopsis *sbt5.2* mutant exhibited enhanced resistance against the bacterial activity (Serrano et al., 2016). TomatoSlSBT69B and SlSBT69C were shown involving in resistance to bacterial wilt *Ralstonia solanacearum*, functioning as pathogenesis-related (PR) proteins and being activated by both pathogen infection (*Phytophothora infestans*) and SA (Zhang et al., 2024; López-Gresa et al., 2016; Christ and Mösinger, 1989; Norero et al., 2016). It is intriguing to observe the involvement of SBT-SA mediated signaling in regulating pathogen attack in both Arabidopsis and tomato studies (AtSBT3.3 and Sl69). Additionally, a soybean-derived SBT peptide, GmSubPep (Glyma18g48580), was shown to induce defense-related genes such as Cyp93A1, Chib-1b, and PDR12 (Pearce et al., 2010). However, the functional characterization of GmSubPep remains unexamined. Moreover, knocking down TaSBT1.7 in wheat using barley stripe mosaic virus-induced gene silencing compromised the hypersensitive response and resistance against *Puccinia striiformis* f. sp. *tritici*, the causal agent of wheat stripe rust (Yang et al., 2021). Grapevine expanded sixteen SBTs as the arsenal for immune signaling, homologous to AtSBT3.3 and SlP69C, at near the Resistance to *Plasmopara viticola* (RPV) locus. Among these sixteen genes, three specific SBT genes, XM_002278414.3 (homologous to AtSBT3.3), XM_002275435.2 (highly homologous to SlP69C), and XM_010660203.1 (annotated as VvCucumsin), showed elevated expression levels against *P. viticola* in grapevine. This suggests that these three genes may contribute to the defense responses of resistant genotypes (Norero et al., 2016; Figueiredo et al., 2016). Given that, plants continually interact with various microbes in their natural environment. These identified subtilases present a valuable opportunity to devise plant-pathogen responses. Leveraging these peptides could improve pathogen resistance in vulnerable crops under climate changes, especially Solanaceae.

Using AtSBT3.3, SlP69B, SlP69C, and VvCucumsin as references, three CaSBTs-CA03g21240, CA01g03850, and CA01g03840 were identified as orthologs in pepper (**Supplementary Table S2**). Although the role of AtSBT5.2 in defense signaling is not fully understood, this subtilase is hypothesized to regulate the transcription of defense-related genes (Serrano et al., 2016). Thus, CRISPR-based editing studies targeting key SBTs, AtSBT3.3, AtSBT3.5 and AtSBT5.2 in *Arabidopsis*, SlSBT69B and SlSBT69C in tomato and CaSBTs (CA03g21240, CA01g03850, and CA01g03840) in pepper could help clarify their function in plant immunity. Priority should be given economically important Solanaceae crops like tomato and pepper, which are particularly vulnerable to pathogen attacks under climate changes (Poulicard et al., 2024; Ma et al., 2023) (**Supplementary Table S2**).

## SBTs in fruit development and ripening

The proteolytic activities of SBTs were tentatively associated during fruit development and ripening, although not thoroughly investigated (Othman and Nuraziyan, 2010). A study on CTG134, a precursor of RGF/GLV (GOLVEN-like) peptide hormones, highlights the role of SBT6.1 in ethylene-auxin mediated peach ripening. However, the subtilase responsible for cleaving the GOLVEN-like peptide CTG134 (DYSPARRKPPIHN) remains unidentified (Tadiello et al., 2016) (**Figure 1B**). AtSBT3.5 cleaved a cell wall pectin methyltransferase (AtPME17) pro-peptide, which activated AtPME17 and enabled its secretion by targeting pectin methylesterase inhibitor (PMEI) domains at a conserved ‘R(R/K)LL’ processing site (Sénéchal et al., 2014, 2015). Similarly, CaPMEI1, the pectin methylesterase inhibitor gene isolated from pepper, is induced by exogenous ethylene and methyl jasmonate treatments (An et al., 2009). A recent study reported that Arabidopsis *sbt3.3* and *sbt3.5* showed a reduced PME activity (Coculo et al., 2023). Thirteen of SlPME orthologs among 57 SIPME cell wall proteins in tomato accumulate significantly during fruit ripening (Wen et al., 2020). Thus, the proteolytic activity of SBTs is possibly relevant to fruit ripening. This prompts the question: could PME function as a peptide hormone in tomato or pepper fruit ripening? Solanaceae genome databases showed strong homology of AtPME17 with tomato Solyc01g091050.1, pepper CA06g06390, and tobacco XP_016471127.1. If so, does SBT3.5 cleave proPME(s) in tomato or pepper, or might another SBT be involved? Therefore, CRISPR-mediated editing of SlSBT3.5 (Solyc07g041970.2) in tomato and CaSBT3.5 (CA03g21240) in pepper would be essential to further investigate SBTs’ role in fruit ripening (**Supplementary Table S2**).

## SBTs in modulating root growth and architecture

Another intriguing aspect of SBTs is their involvement in cleaving peptides involved in root growth, particularly the GOLVEN (GLV) peptide, which play a critical role in several plant developmental processes like root development and nodule elongation (Roy et al., 2024; Stegmann et al., 2022). Through a genetic suppressor screening in Arabidopsis, AtSBT6.1 (AT5G19660) and AtSBT6.2 (AT4G20850) were identified as essential factors for GLV1 activity in root cell elongation (Ghorbani et al., 2016, 2015). *In vitro* studies showed that synthetic GLV-derived peptides were cleaved by the affinity-purified AtSBT6.1 subtilase, confirming GLV1 as a direct SBT substrate. Additionally, mutating the *in vitro* SBT recognition sites through alanine substitution, suppressed the GLV1 gain-of-function phenotype *in vivo*. The protease inhibitor serpin1 was found to bind to AtSBT6.1, inhibiting the cleavage of GLV1 precursors (Ghorbani et al., 2016). Another study on AtSBT6.1 is also involved in the cleavage of CLEL1 and CLEL6 pro-peptides (root growth factors) (Stührwohldt et al., 2020). These two studies demonstrated that the active role of GLV1 and CLEL peptides in root growth is dependent on SBTs, specifically AtSBT6.1. Considering the retarded root growth observed in Arabidopsis *sbt6.1* mutant, investigation of the loss of function effect of SBT6.1 on root development in Solanaceae crops, particularly pepper would be imperative for understanding of root development. A homology search identified a putative SBT6.1 ortholog in pepper, CaSBT6.1 (CA09g03290) (**Figure 1B**) (**Supplementary Table S2**).

## Roles of SBTs in seed development and germination

Plant SBTs are involved in seed development by mediating key peptide activation. For instance, AtSBT1.8 (AT2G05290), exhibited a crucial role in cleaving the proTWS1 peptide to generate the active TWS1 (Twisted Seed1) peptide (Royek et al., 2022). TWS1, identified as a novel small peptide is essential for the seed development process (Fiume et al., 2016). Similar to many other SBT studies, AtSBT1.8 was also identified through a cleavage assay (Royek et al., 2022). However, knocking out or over-expression of AtSBT1.8 have not been performed to date. Interestingly, a homology search for AtTWS1 (AT5G01075) revealed a high degree of similarity (71%) to an uncharacterized gene, CaTWS1 (CA04g17950) only in pepper, with no significant hits in tomato, tobacco, or potato genome. This raises a question about the role of CaSBT1.8 (CA07g06400) in cleaving CaTWS1, as the alignment results suggest a similar cleavage site for both AtTWS1 and CaTWS1 peptides (**Figure 1C**). Therefore, both AtSBT1.8 and CaSBT1.8 targeted editing could provide valuable insights for improving seed development processes in other Solanaceae crops. Selecting optimal seed size and viability is an essential trait for crop improvement (**Figure 1C**).

The role of SBTs in seed germination has only a few documented reports in recent years. Three barley SBTs, AK355289, AK362004, and AK361952, showed high expression during seed germination stages and were identified through cleavage assay (Galotta et al., 2019), but their impact on barley germination remains unknown. Furthermore, studies on SBTs in seeds have been reported for *Arabidopsis*, soybean, barley and rice, however many of these studies are more than a decade old; AtSBT1.7 was involved in the release of mucilage from the seed coat during rehydration (Rautengarten et al., 2008) and AtALE1 (Abnormal Leaf Shape 1) controls embryo cuticle formation (Yang et al., 2008). A previous study on the model legume *Medicago truncatula* identified MtSBT1.1 involved in the regulation of cotyledon cell number, rather than cell expansion, during seed development (D’Erfurth et al., 2012). Since seeds of legumes, such as pea and soybean, are rich sources of proteins for both animal and human nutrition, understanding the molecular mechanisms regulating seed development is crucial for developing strategies to improve seed quality and yield. Interestingly, a homologous search for pepper using AtSBT1.7 (AT5G67360.1) and MtSBT1.1 (AES94589.1) resulted in a single candidate, CaSBT1.7 (CA02g25020) (**Supplementary Table S2**). The role of potential CaSBT1.7 in pepper seed development and germination remains to be assessed using CRISPR tool, thus the positive correlation could have a significant impact on seed development in economically important horticultural crops.

## Functions of SBTs in plant abscission and organ separation

Although plant organ abscission is a natural process, it has become a significant yield-reducing factor in horticultural crops, especially under stress conditions (Li and Su, 2024). The role of SBTs in plant abscission was recently demonstrated in two notable studies on *Arabidopsis* and tomato (Reichardt et al., 2020; Stührwohldt et al., 2018). Organ abscission (like flowers, petals, and sepals) in *Arabidopsis* and drought stress-induced flower drop in tomato were both mediated by SBTs, though the players and mechanisms involved differed between species. AtSBT4.12, AtSBT4.13, and AtSBT5.2, cleaved the proIDA (Inflorescence Deficient in Abscission) peptide, subsequently leading to the formation of mIDA (the mature and the bioactive form of IDA) as the endogenous signaling peptide required for the floral organ abscission (Schardon et al., 2016). The tomato caspase-like protease enzyme Phytaspase2 (SlPhy2), a unique subtilase subtype due to its extremely high substrate specificity and known for hydrolyzing peptide bonds immediately after cleaving at the ‘Asp’ residue, has been shown to cleave the proPSK (phytosulfokine) peptide, thereby mediating abscission. This function parallels the activity of SlSBT3.8, which cleaves proPSK to enhance drought stress tolerance (Reichardt et al., 2020; Schardon et al., 2016; Stührwohldt et al., 2018, 2021; Chichkova et al., 2018). Tomato mutant *phy2* prevented floral drop, clearly establishing a function in organ abscission (Reichardt et al., 2020), which indicated that SlPhy2 might perform similar function in other Solanaceae crops (**Figure 1D**).

However, the knowledge of Phy2 gene in other Solanaceae crops including pepper is currently unknown. Phylogenetic analysis revealed that Phy2 genes have distinct evolutionary origins, with NtPhy2 (LOC107789361) and CaPhy2 (CA04g18510) diverging earlier than SlPhy2, yet Solanaceae shared highly conserved regions at the peptide activation site (GTSMSCPHASG) (data not shown; **Supplementary Table S2**). This suggests that the identified CaPhy2 and NtPhy2 might have potentially similar function to SlPhy2. Additionally, pepper have six paralogs, CaPhy1 (CA12g16680), CaPhy2 (CA04g18510), CaPhy3 (CA04g18500), CaPhy4 (CA12g16690), CaPhy5 (CA12g16700) and CaPhy6 (CA06g21680), based on homologous comparisons with six-tomato phytaspase (**Supplementary Table S1**). However, the functional characterization of ‘Phy’ genes in pepper and other Solanaceae remain unknown. Furthermore, flower abscission remains a longstanding issue in horticultural crops, particularly Solanaceae such as tomato and pepper, especially under stress conditions (Riga, 2014; Shi et al., 2023).

Flower abscission significantly reduces fruit yield, leading to substantial losses for farmers (Tonutti et al., 2023). Given the role of phytaspases in cleaving signaling PSK peptides and their importance in stress-induced abscission in tomato and Arabidopsis, both single and multiple (double/triplet/quadruple) gene knockout studies needs to be conducted in closely related Solanaceae crops to further elucidate their role in organ abscission. Further, to provide molecular insight into the pepper SBTs, tertiary peptide structures of CaPhy2 and CaSBT1.7 including its active binding site, is presented (**Figures 1E, F**).

## Conclusion and future perspectives

In summary, SBTs play a pivotal role in plant immunity and growth. These proteases activate networks of multiple signaling pathways and regulate peptide hormones, influencing various physiological processes. Further identification and characterization of novel SBTs could offer promising strategies to modulate their activity using molecular techniques such as CRISPR, enabling precise responses, particularly in Solanaceae family. Currently, few studies focus on the molecular changes in plant tissues following SBT gene knockout or over-expression. Expanding such research to a broader range of horticultural crops could deepen our understanding of the complex networks influencing key agronomic traits, including yield and morphological features. The recent discovery of a subtilase subtype, ‘phytaspase,’ holds significant potential for mitigating flower drop under stress conditions, thereby enhancing crop yield in economically important plants. Additionally, the application of advanced gene-editing tools could facilitate more targeted breeding programs, resulting in crops with improved immunity and better adaptation to evolving climate conditions. This mini-review provides new insights into predicted SBT peptides in pepper, a vital Solanaceae crop, which could serve as a foundation for future research and applications.
