# Supplementary material for: Subtilases: a major prospect to the genome editing in horticultural crops
Source: Front Plant Sci. 2025 Jan 7;15:1532074. doi: 10.3389/fpls.2024.1532074 (PMC11752874; doi:10.3389/fpls.2024.1532074)
Supplement: Supplementary file 3 [file Table3.doc]

**
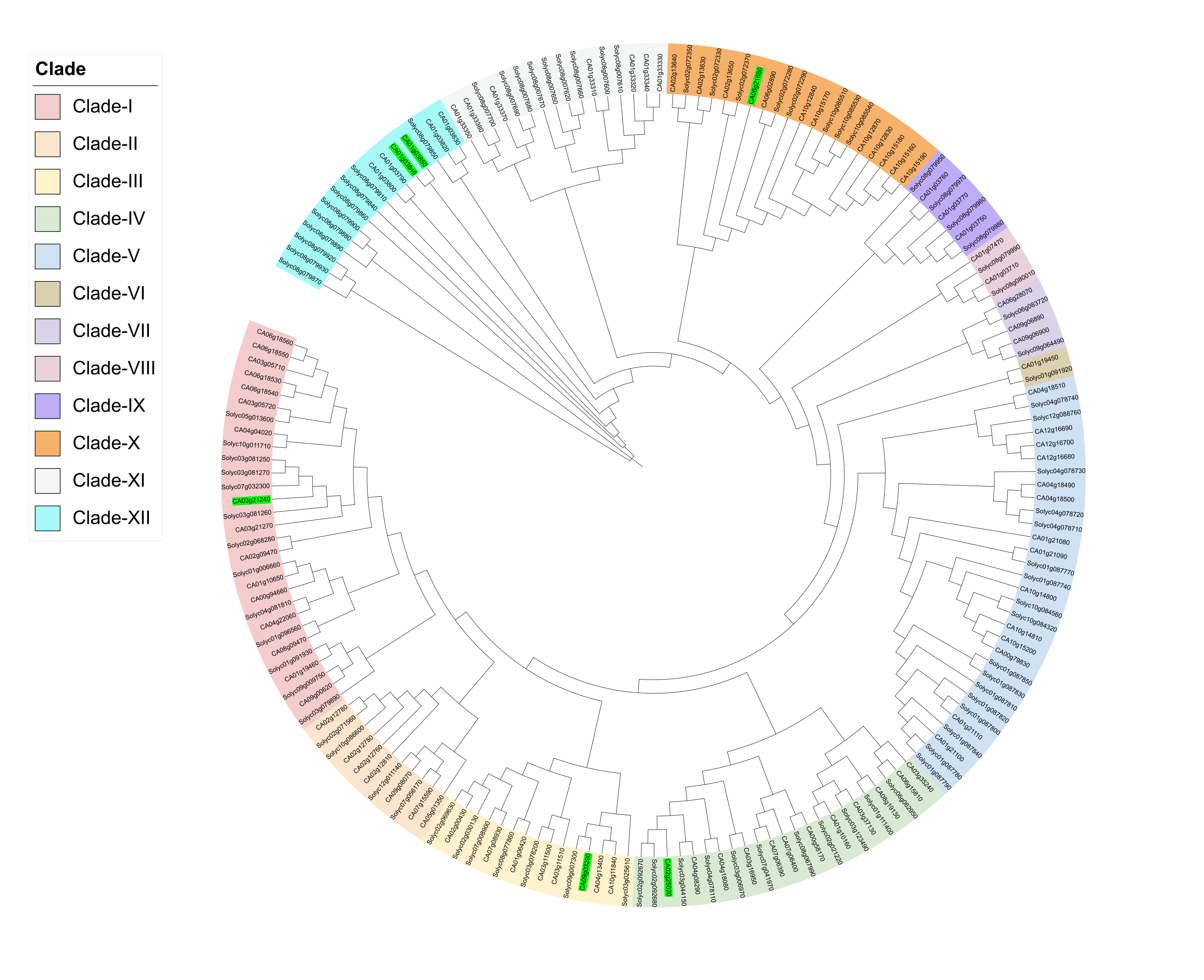
**

**Figure S1.** **Phylogenetic analysis of SBTs in tomato and pepper.** Genome sequence information (ITAG4.1 for tomato and CM334 for pepper) was retrieved from the Sol Genomics Network (https://solgenomics.net/) to elucidate the SBT genes in tomato and pepper. After retrieving the information, we annotated the Pfam domain using InterProScan software (version 5.66-98.0). As a result, 88 and 91 genes containing the subtilase domain (PF00082) were identified in tomato and pepper, respectively. The Protein sequences of these 179 SBT genes were aligned using MAFFT software (version 7.520) and a phylogenetic tree was generated with FastTree (version 2.1.11) using 1000 bootstrap tests. Identified SBTs were designated as 12 clusters (from Clade I to Clade XII). Clade I and Clade V each contained fourteen CaSBTs with clade VI containing only one CaSBT. Immune responsive pepper subtilases like CA03g21240, CA01g03840 and CA01g03850 were distributed in Clade I and Clade XII respectively. Possible candidate involved in pepper root development (CA09g03290) and seed development (CA02g25020) were placed in Clade III and Clade IV, respectively. Five out of six phytaspases (Phy) identified in pepper were grouped in Clade V grouped with their respective ortholog identified from tomato with only one CA06g21680 distributed in clade X.
